# Supplementary material for: Distinct subdivisions of human medial parietal cortex support recollection of people and places
Source: eLife. 2019 Jul 15;8:e47391. doi: 10.7554/eLife.47391 (PMC6667275; doi:10.7554/eLife.47391)
Supplement: Figure 6—figure supplement 1—source data 1. [file elife-47391-fig6-figsupp1-data1.zip › Figure6-figure supplement 1-source data/README.rtf]

#### Supplementary Data File 4 Silson et al. ####The Supplementary Data is a matlab readable .mat matrix with the following dimensions:SILSONETAL.data4:This matrix has 4-dimensions [participants(1-24), hemispheres(lh, rh), rois(PPA, FFA), Conditions(Famous People, Famous Places, Personal People, Personal Places)].Each cell represents the mean t-value vs baseline, for that participant, hemisphere, row and condition.
