# Supplementary material for: Distinct subdivisions of human medial parietal cortex support recollection of people and places
Source: eLife. 2019 Jul 15;8:e47391. doi: 10.7554/eLife.47391 (PMC6667275; doi:10.7554/eLife.47391)
Supplement: Figure 7—source data 1. [file elife-47391-fig7-data1.zip › Figure7-source data 1/README.rtf]

#### Supplementary Data File 3 Silson et al. ####The Supplementary Data is a matlab readable .mat matrix with the following dimensions:SILSONETAL.data3:This matrix has 4-dimensions [participants(1-24), hemispheres(lh, rh), rois(ROI1, ROI2, ROI3, ROI4), Conditions(Famous People, Famous Places, Personal People, Personal Places)].Each cell represents the mean t-value vs baseline, for that participant, hemisphere, row and condition. This value was derived by first defining each ROI in the Odd data and sampling the values from the Even data. This process was then reversed and the averaged computed.
