# Supplementary material for: Distinct subdivisions of human medial parietal cortex support recollection of people and places
Source: eLife. 2019 Jul 15;8:e47391. doi: 10.7554/eLife.47391 (PMC6667275; doi:10.7554/eLife.47391)
Supplement: Figure 7—figure supplement 1—source data 1. [file elife-47391-fig7-figsupp1-data1.zip › Figure7-figure supplement 1- source data 1/README.rtf]

#### Supplementary Data File 6 Silson et al. ####The Supplementary Data is a matlab readable .mat matrix with the following dimensions:SILSONETAL.data6:This matrix has 4-dimensions [participants(1-65), hemispheres(lh, rh), Seed(MPCv, MPCd), Eccentricity(Foveal, Peripheral)].Each cell represents the mean functional connectivity between pairs of ROIs.
