# Supplementary material for: Distinct subdivisions of human medial parietal cortex support recollection of people and places
Source: eLife. 2019 Jul 15;8:e47391. doi: 10.7554/eLife.47391 (PMC6667275; doi:10.7554/eLife.47391)
Supplement: Transparent reporting form [file elife-47391-transrepform.docx]

***eLife’s* transparent reporting form**

We encourage authors to provide detailed information *within their submission* to facilitate the interpretation and replication of experiments. Authors can upload supporting documentation to indicate the use of appropriate reporting guidelines for health-related research (see [EQUATOR Network](http://www.equator-network.org/%20)), life science research (see the [BioSharing Information Resource](https://biosharing.org/)), or the [ARRIVE guidelines](http://www.plosbiology.org/article/info:doi/10.1371/journal.pbio.1000412) for reporting work involving animal research. Where applicable, authors should refer to any relevant reporting standards documents in this form.

If you have any questions, please consult our Journal Policies and/or contact us: [editorial@elifesciences.org](mailto:editorial@elifesciences.org).

**Sample-size estimation**

- You should state whether an appropriate sample size was computed when the study was being designed
- You should state the statistical method of sample size computation and any required assumptions
- If no explicit power analysis was used, you should describe how you decided what sample (replicate) size (number) to use

Please outline where this information can be found within the submission (e.g., sections or figure legends), or explain why this information doesn’t apply to your submission:

The sample size for both the resting-state functional connectivity analysis (n=65) and the analysis of visual stimulation (n=29) were convenience samples, as these data had already been collected as part of prior projects from our group. The sample size for the memory task (n=24) was determined based on a prior fMRI study of memory-recall from our group employing a very similar paradigm (e.g. Silson et al., 2019). This information is specified under Materials and *Methods > Participants.*

**Replicates**

- You should report how often each experiment was performed
- You should include a definition of biological versus technical replication
- The data obtained should be provided and sufficient information should be provided to indicate the number of independent biological and/or technical replicates
- If you encountered any outliers, you should describe how these were handled
- Criteria for exclusion/inclusion of data should be clearly stated
- High-throughput sequence data should be uploaded before submission, with a private link for reviewers provided (these are available from both GEO and ArrayExpress)

Please outline where this information can be found within the submission (e.g., sections or figure legends), or explain why this information doesn’t apply to your submission:

A technical replication was built into the analysis of the memory experiment. Specifically, we separated the memory 6 fMRI runs into two halves (odd, even). These halves were then analyzed separately, and the results compared using Pearson’s correlation coefficient. This split-half analysis demonstrated the highly reliable and replicable nature of the memory effects were report. The exact method used to perform this split-half analysis is reported in *Materials and Methods > Split-half analysis* and the resulting Pearson’s r values are reported in *Results*.

Criteria for inclusion in the resting-state analysis was that each participant completed a minimum of 20 minutes of resting-state fMRI data.

Criteria for inclusion in functional localizer analysis was that each participant completed all six runs of the functional localizer experiment.

Criteria for inclusion in the memory experiment was that each participant completed all six runs of the memory experiment.

**Statistical reporting**

- Statistical analysis methods should be described and justified
- Raw data should be presented in figures whenever informative to do so (typically when N per group is less than 10)
- For each experiment, you should identify the statistical tests used, exact values of N, definitions of center, methods of multiple test correction, and dispersion and precision measures (e.g., mean, median, SD, SEM, confidence intervals; and, for the major substantive results, a measure of effect size (e.g., Pearson's r, Cohen's d)
- Report exact p-values wherever possible alongside the summary statistics and 95% confidence intervals. These should be reported for all key questions and not only when the p-value is less than 0.05.

Please outline where this information can be found within the submission (e.g., sections or figure legends), or explain why this information doesn’t apply to your submission:

The fMRI data was preprocessed using standard procedures using publicly available software (AFNI).

For statistical tests comparing the responses evoked by visual presentation of different visual categories in each ROI we performed a one-way repeated measures ANOVA with Category (6 levels) as a within-participant factor. If a significant main effect of Category was observed, we performed pair-wise comparisons using Bonferroni correction. We include the exact p-value and partial eta^2^ estimates in *Results > Subdivisions of MPC show differential responses to visually presented categories,* and full statistical breakdown of all main effects and interactions is reported in *Supplementary Material.* Further, we plot the mean response, as well as each individual participant data point in Figure 2.

The same statistical approach was adopted when comparing the responses evoked during memory recall from all ROIs, whether defined using resting-state, via the split half-analysis, category-selectivity or anatomical selection. In each case, the mean response to each condition (Famous people, Famous places, Personal people, Personal places) was calculated in each participant. These values were then subjected to a three-way repeated measures ANOVA with Category (People, Places), Familiarity (Famous, Personal) and Hemisphere (Left, Right) as within-participant factors. If a significant three-way interaction was observed, we further explored the nature of this interaction with two-way ANOVAs in each hemisphere separately. We include the exact p-value and partial eta^2^ estimates in *Results,* and full statistical breakdown of all main effects and interactions is reported in *Supplementary Material.* Further, we plot the mean response, as well as each individual participant data point in Figures 4 & 7 and Supplementary Figures 2, 3, & 4.

(For large datasets, or papers with a very large number of statistical tests, you may upload a single table file with tests, Ns, etc., with reference to sections in the manuscript.)

**Group allocation**

- Indicate how samples were allocated into experimental groups (in the case of clinical studies, please specify allocation to treatment method); if randomization was used, please also state if restricted randomization was applied
- Indicate if masking was used during group allocation, data collection and/or data analysis

Please outline where this information can be found within the submission (e.g., sections or figure legends), or explain why this information doesn’t apply to your submission:

There was only one experimental group per experiment, thus group allocation was unnecessary.

**Additional data files (“source data”)**

- We encourage you to upload relevant additional data files, such as numerical data that are represented as a graph in a figure, or as a summary table
- Where provided, these should be in the most useful format, and they can be uploaded as “Source data” files linked to a main figure or table
- Include model definition files including the full list of parameters used
- Include code used for data analysis (e.g., R, MatLab)
- Avoid stating that data files are “available upon request”

Please indicate the figures or tables for which source data files have been provided:

To facilitate replicability and transparency, the numerical data making up the bar plots for 1) response in MPC to visually presented stimuli 2) the response in MPC during memory recall 3) the split-half response in MPC during memory recall 4) the response in VTC during memory recall 5) the response in subcortical structures during memory recall and 6) the resting-state connectivity between MPC and EVC (i.e. the source data for the analyses reported in Figures 2, 4 & 7, Supplementary Figures 2, 3 & 4) are provided as Supplementary Data.
